# Assessment of the clinical and laboratory risk factors for thrombosis in neonates admitted to neonatal intensive care unit (two Egyptian tertiary centers experience)

**DOI:** 10.1007/s00277-024-06002-5

**Published:** 2024-10-01

**Authors:** Ebtihal Mokhtar Abdelsamei, Gehan Lotfy Abdel Hakeem, Nadia Mohamed El Amin, Maha Ahmed Yousef, Hager Samy Ghalioub, Zamzam Hassan Mohamed

**Affiliations:** 1 Pediatric Department, Minia University, Al Minya, Egypt; 2 Radiology Department, Minia University, Al Minya, Egypt; 3 Pediatric Department, Alexandria University, Alexandria, Egypt

**Keywords:** Neonatal intensive care unit (NICU), Neonatal thrombosis, Risk factors, Outcomes

## Abstract

In neonates admitted to the neonatal intensive care unit (NICU), arterial and venous thromboembolism is a major cause of morbidity and death which could be attributed to multiple risk factors exposure. This study aimed to evaluate the clinical characteristics, laboratory and radiological assessments, predisposing risk factors, and outcomes of thrombosis in neonates admitted to NICU. This prospective cohort study was conducted at NICU, Minia, and Alexandria University Children’s Hospital. Screening of 886 patients admitted to NICU over one year with different clinical presentations, patients were classified into the thrombotic and non-thrombotic groups based on the presence or absence of thrombosis. Thrombosis was diagnosed based on clinical, laboratory and different radiologic assessments. Genetic testing for factor V Leiden mutations G1691A, prothrombin mutation G20210A, protein C, protein S, and antithrombin III gene mutations were performed for patients with a family history of thrombosis. Out of a total of 886 neonatal admissions, 36 patients were diagnosed with evident thrombosis (40 per 1000 NICU admissions). The sites of venous thrombosis detection were Portal vein thrombosis in 11 patients (30.6%), superior vena cava thrombosis in 7 patients (19.4%), deep venous thrombosis in 5 patients (13.9%), central venous thrombosis in 5 patients (13.9%), intra-cardiac thrombosis in 3 patients (8.3%) and necrotic skin patches in one patient (2.8%). Only 69% of enrolled thrombosis patients showed genetic mutations the most common of which was factor V Leiden mutation (52.3%). Sepsis, central venous line (CVL) insertion, C reactive protein (CRP), and duration of NICU admission were significantly more common in the thrombotic group (*p* < 0.001) and were associated with a higher risk of thrombosis (ORs: 1.02, 7.7, and 1.11, respectively) (*p* < 0.001). Higher mortality occurred in thrombosis neonates compared with a non-thrombotic group (52.8% versus 17.4%) (*p* < 0.001). NICU-admitted neonates are exposed to multiple overlapped risk factors, the detection of which is important for preventing potential thrombosis and improving the patient’s outcomes. The complexity of sepsis pathogenesis and management could potentiate multiple acquired risk factors. inherited thrombophilia detection is required for prevention of further morbidities.

## Introduction

Critically ill newborns have a significant risk of thrombosis when compared to older children [1]. Decreased concentrations of procoagulant proteins, naturally occurring anticoagulants, and hemostatic control proteins cause an imbalance between prothrombotic and antithrombotic processes in the neonate’s developing hemostatic system. Overall, neonatal hemostasis tends to be a prothrombotic state [2, 3]. Venous and arterial thrombosis are important causes of morbidity and mortality in neonates admitted to neonatal intensive care units (NICUs) [4]. Approximately 2–4% of infants with thrombosis die as a direct result of thrombosis [4–6], and mortality in infants with thrombosis is as high as 33% [7]. End-organ damage and limb-threatening morbidity are associated with renal vein thrombosis, portal vein thrombosis, femoral arterial thrombosis, pulmonary embolus, and hepatic artery thrombosis [4, 6, 8, 9].

Factors associated with neonatal thrombosis are central access devices (CADs) and prematurity in addition to other maternal and neonatal risk factors. (e.g. perinatal asphyxia, maternal diabetes, polycythemia, septicemia, poor cardiac output, and dehydration) [10–13]. Neonatal risk factors includ male sex, bloodstream infection, and prolonged mechanical ventilation [2, 14, 15]. The approach to managing neonatal thrombosis can be observation versus treatment with anti-thrombotic therapies. This depends on the location, occlusive nature, and underlying co-morbidities [16]. The diverse forms of thrombotic illness in neonates, the various signs and symptoms of thrombosis, and difficulties in predicting thrombosis occurrence have made the study of risk factors challenging. Additionally, no previous study regarding this concern in our locality. In this study, we aimed to evaluate the clinical characteristics, the laboratory assessments, the predisposing risk factors, and outcomes of thrombosis in neonates admitted to NICU.

## Patients and methods

### Study population

This prospective cohort study was conducted at NICU in Minia-University Children’s Hospital and El Shatby Children’s Hospital over one year from March 2023 to February 2024. All neonates admitted to the NICU during this interval were approached and all studied neonates were classified into 2 groups:

Thrombotic group: Neonates who proved to have thrombosis (from birth to 28 days, full-term and preterm diagnosed with clinically suspected thrombosis confirmed by the imaging technique.)

Non-thrombotic group: all neonates from birth to 28 days were admitted to NICU during the study period with no clinical or radiologic evidence of thrombosis. Patients with severe congenital anomalies were excluded from the study.

The patients with confirmed thrombosis were subjected to detailed medical history (perinatal/neonatal, gestational age, gender, birth weight, maternal obstetric history, family history of thrombophilia or thromboembolic events, the cause of admission to NICU, site of thrombus and associated co-morbid conditions [sepsis, mechanical ventilation, vascular access], duration of vascular access either peripheral or central, treatment received [FFP, Thrombolytics drugs] and its duration, duration of NICU admission, and the survival status at discharge. Thorough clinical examination included general, cardiac & chest, abdominal, and neurological examination.

### Laboratory assessment

Complete blood count (CBC), C-reactive protein (CRP), Prothrombin concentration and time, activated Partial thromboplastin time (aPTT), liver function test, renal function test, and serial blood cultures were also collected for all enrolled patients.

Patients with proven thrombosis were subjected to thrombophilia genetic screening according to the standard (Antithrombin III, Protein C, or protein S) plasma activity, factor V Leiden mutation G1691A, and prothrombin mutation G20210A gene mutation [17].

### Sampling

Eight ml of venous blood samples were withdrawn from all patients. The sample was divided into (1) 1 ml in a sterile vacutainer tube containing EDTA solutions for CBC assay by automated cell analyzer (Nihon Kohden Europe). (2) 2 ml on EDTA solutions for DNA isolation and further assessment of genetic detection (QiaAmp DNA extraction kit, Qiagen). By spectrophotometer (Thermofisher Scientific, USA); (3) 1.8 ml of blood on.

0.2 ml trisodium citrate for measuring PT and PTT (4) 3 ml of venous blood for assessing the renal and liver function tests, CRP (Shenzhen Genius Electronics, China). by the automated chemistry auto-analyzer system (Selectra proM, ELITech Group, Finland).

FV Leiden and prothrombin gene mutations were analyzed using reversed hybridization assay strips. For each polymorphic position, three patterns were obtained: homozygous normal, heterozygous, or homozygous mutant genotype.

### Radiological assessment

Color duplex study for examining the state of most body vessels, mainly those peripheral vessels at the upper and lower limbs. It is also utilized for detecting carotid vessels and main mesenteric vessels. Doppler ultrasound on portal circulation was used to detect portal vein thrombosis. Skin necrosis is detected by the naked eye examination as bluish to blackish skin patches with decreased blood flow to the affected area, confirmed by superficial ultrasound on the affected site.

Echocardiography was used to diagnose intracardiac thrombosis and to confirm Superior vena cava thrombosis (SVC).

Brain magnetic resonance imaging (MRI), magnetic resonance arteriography (MRA), magnetic resonance venography (MRV), and/or computed tomography and angiography were used to diagnose central nervous system (CNS) thrombosis.

### Statistical analysis

All data were collected, tabulated, and statistically analyzed using SPSS 26 for Windows (SPSS Inc., Chicago, IL, USA). Data were tested for normal distribution using the Shapiro Walk test. Qualitative data were represented as frequencies and relative percentages. The chi-square test (χ2) and Fisher exact were used to calculate the difference between qualitative variables as indicated. Quantitative data were expressed as mean ± SD (Standard deviation) and median with range for parametric and non-parametric data. Independent T-test and Mann-Whitney test were used to calculate the difference between quantitative variables in two groups for parametric and non-parametric variables, respectively. Univariate and multivariate regression analysis was done for the determination of risk factors associated with thrombosis. All statistical comparisons were two-tailed with a significance level of *p*-value ≤ 0.05, indicating a significant difference, and *p* < 0.001, indicating a highly significant difference.

## Results

Out of 886 neonatal admissions to our NICU between the 1st of February 2023 and the 31st of January 2024, 36 patients had thrombosis (thrombotic group) and 850 were thrombosis free (non-thrombotic group), The incidence of thrombosis was estimated to be 40 per 1000 NICU admissions. Three hundred fifty (35.55%) patients were admitted with respiratory distress, 287 (32.39%) with neonatal jaundice, 90 (10.15%) were preterm, 88 (9.93%) were infants of diabetic mothers (IDM), 46 (5.19%) with sepsis, 10 (1.12%) with convulsions and 50 (5.64%) patients were admitted with other causes.

The mean age of the 36 patients diagnosed with thrombosis was 8.3 (4.4) days. The mean (SD) gestational age was 36.3 (1.5) weeks. 55.6% were male. 55.6% were delivered by cesarean section, and 44.4%were delivered by normal vaginal delivery with a mean birth weight of 1900 g. Neonates with thrombosis had significantly lower birth weight (*p* = < 0.001) than non-thrombotic ones. There were no significant differences in gender, family history of thrombophilia, mode of delivery, or gestational age between the thrombosis and non-thrombotic patients. Premature infants represented 58.3% of the thrombotic cases (Table 1). The thrombotic patients showed a significantly higher duration of NICU admission compared to the non-thrombotic ones (21.4 ± 10.2 vs. 7.5 ± 6.8 days, *p* < 0.001). regarding the causes of NICU admission in thrombotic cases, respiratory distress was present in 11 patients (30.6%), prematurity in 8 patients (22.2%), neonatal sepsis in 5 patients (13.9%), jaundice in 4 patients (11.1%), 3 patients (8.3%) were infants of diabetic mothers, convulsion in 2 patients (2.7%), and 3 other patients were admitted due to other causes. The majority of the thrombotic patients (23 cases) (63.9%) had no history of maternal risk factors of medical importance.

**Table 1:** Demographic data of thrombotic and non-thrombotic groups

Baseline characteristic/cs	Thrombotic group (*n* = 36)	Non-thrombotic group (*n* = 850)	*p*-value
Mean age of onset of thrombosis (days)	8.3 ± 4.4	-----------	
Gender Male/ Female	20(55.6%)/16(44.4%)	489(57.5%)/ 361(42.5%)	0.81
Mode of delivery Normal/ CS	16(44.4%)/20 (55.6%)	489(57.5%)/361(42.5%)	0.12
Family history Positive/ Negative	7(19.4%)/29(80.5%)	90(10.6%)/760(98.4%)	0.10
Mean birth weight(gm)	1.9 ± 0.47	2.5 ± 0.7	< 0.001*
Mean gestational age (week)	36.3 ± 1.5	36.9 ± 1.8	0.15
Prematurity < 37 week	21(58.3%)	376 (44.2%)	0.9

CS = cesarean section, * significant at *p*-value < 0.05

Five patients (13.9%) were associated with maternal hypertension, 3 (8.3%) had a history of maternal diabetes mellitus, and premature rupture of membranes (PROM) in mothers was found in 2 patients (5.6%).

There was a statistically significant difference between the two studied groups as regards the number of inserted vascular accesses (*p* < 0.001). Single vascular access insertion was associated with a decreased risk of neonatal thrombosis (*p* = 0.004). Central venous line insertion was more common among thrombotic patients compared to non-thrombotic ones (38.9% vs. 35%; *p* < 0.001).

A significantly higher incidence of sepsis was found among thrombotic patients than in non-thrombotic cases (69.4% vs. 35.8%; *p* < 0.001) while non-significant differences between both groups of patients regarding the age of NICU admission (days), caused of NICU admission, need for mechanical ventilation, and the presence of maternal risk factors (*p* < 0.001). Higher mean total leukocytic count and C reactive protein were found in thrombotic patients than in non-thrombotic ones (*p* < 0.001) (Table 2).

**Table 2:** Some clinical and laboratory data between the thrombotic group and non-thrombotic group

Clinical and laboratory data		Thrombotic group (*n* = 36)	Non-thrombotic group (*n* = 850)	*P* value
Mean age of NICU admission(days)		3.6 ± 3.6	3.4 ± 5.4	0.84
Mean Duration of admission(days)		21.4 ± 10.2	7.5 ± 6.8	< 0.001*
Cause of NICU admission	Respiratory	11(30.6%)	304(35.8%)	0.3
	Jaundice	4 (11.1%)	283(33.3%)	
	Prematurity	8(22.2%)	82(9.6%)	
	Convulsion	2(2.7%)	8(0.9%)	
	Sepsis	5(13.9%)	41(4.8%)	
	IDM	3(8.3%)	85(10%)	
	Other	3(8.3%)	47(5.5%)	
Maternal Risk factors	No history of medical importance	23(63.9%)	546(64.2%)	0.58
	Hypertension	5(13.9%)	77(9.1%)	
	DM	3(8.3%	97(11.4%)	
	PROM	2(5.6%)	90(10.9%)	
	Other	3(8.3%)	40(4.7%)	
Type of vascular access	UVC	8(22.2%)	166(19.6%)	0.69
	CVL	14(38.9%)	35(4.1%)	< 0.001*
	Peripheral line	31(86.1%)	669(78.7%)	0.28
	No vascular access	–	166(19.3%)	0.004*
Number of vascular access No access Single/ Multiple		- 19(52.8%)17(47.2%)	166(19.3% 501(58.9%)/185(21.8%)	< 0.001*
Sepsis No/ Yes		11(30.6%)/25(69.4%)	546(64.2%)/304(35.8%)	< 0.001
Need for mechanical ventilation No/ Yes		22(61%)/14	625(73.5%)/225(26.5%)	0.1
Mean Hemoglobin (g/dl)		12.7 ± 2.9	13.1 ± 2.5	0.25
Mean TLC (×10⁹)		16.6 ± 9.7	9.6 ± 5	< 0.001*
Mean Platelet (×10⁹)		155 ± 88	256 ± 114	0.1
CRP(mg/L) : Negative/ Positive		3(8.3%)/33(91.7%)	546(64.2%)/304(35.8%)	< 0.001*
Mean CRP(mg/L)		50.6 ± 33	14 ± 29.3	< 0.001*

* Significant at *p*-value < 0.05, NICU: neonatal intensive care unit, DM: diabetes mellitus, PROM: premature rupture of membrane, UVC: umbilical venous catheter, CVL: central venous line, TLC: total leucocytic count, CRP: C reactive protein

Portal vein thrombosis, the most common site of thrombosis was detected in 11 patients (30.6%) followed by, SVC thrombosis in 7 patients (19.4%), DVT in 5 patients (13.9%), CNS thrombosis in 5 patients (13.9%), intra-cardiac thrombosis 3 patients (8.3%), necrotic skin patches in one patient (2.8%), and 4 patients (11.1%) had mixed thrombosis in more than one site. (Table 3).

**Table 3:** Sites of thrombosis in the thrombotic group

Diagnosis		Descriptive statistic (*n* = 36)
Site of thrombosis	PVT	11(30.6%)
	DVT	5(13.9%)
	SVC	7(19.4%)
	Intra-cardiac	3(8.3%)
	Skin necrotic patches	1(2.8%)
	CNS	5(13.9%)
	Mixed	4(11.1%)

PVT: portal vein thrombosis, DVT: deep venous thrombosis, SVC: superior vena cava, CNS: central venous system

Univariate analysis showed that sepsis, CVL insertion, CRP, and duration of NICU admission were significant risk factors for thrombosis. (*p* < 0.001). By multivariate logistic regression analysis, CRP, CVL insertion, and duration of NICU admission were independently associated with a higher risk of thrombosis with adjusted ORs of 1.02, 7.7, and 1.11, respectively (*p* < 0.001). (Table 4)

**Table 4:** Univariate and multivariate logistic regression analysis of risk factors associated with thrombosis

Independent Variables			Univariate regression		Multivariate regression	
			OR (95%CI)	*p*-value	Adjusted OR (95%CI)	*p* value
Gestational age		Full term	1	0.10		
		Preterm	1.7(0.89:3.4)			
Gender		Female	1	0.41		
		Male	0.92(0.47:1.8)			
Sepsis		No	1	< 0.001*	1	0.70
		Yes	4(1.9:8.4)		1.2(0.38:4)	
Mechanical ventilation		No	1	0.10		
		Yes	1.7(0.8:3.5)			
Vascular access		UVC	1.17(0.52:1.6)	0.69		
		CVL	14.9(6.9:31.3)	< 0.001*	7.7(3.2:18.9)	0.001*
		Peripheral line	1.7(0.64:4.3)	0.29	1.8(0.5:3.4)	0.4
Maternal risk factors		No history	1			
		DM	0.73(0.21:2.4)	0.62		
		HTN	1.5(0.65:4.1)	0.33		
		PROM	0.52(0.12:2.2)	0.39		
		Other	1.7(0.51:6.1)	0.36		
CRP(mg/L)			1.02(1.01:1.03)	< 0.001*	1.02(1.01:1.03)	0.001*
Duration of NICU admission (days)			1.13(1.9:1.17)	< 0.001*	1.11(1.07:1.15)	0.001*
No of vascular access	Multiple		1			
	Single		0.41(0.21:0.81)	0.01*		

* Significant at *p*-value < 0.05, UVC: umbilical venous catheter, CVL: central venous line, DM: diabetes mellitus, HTN: hypertension, PROM: premature membrane rupture, CRP: C reactive protein

**Fig. 1:**
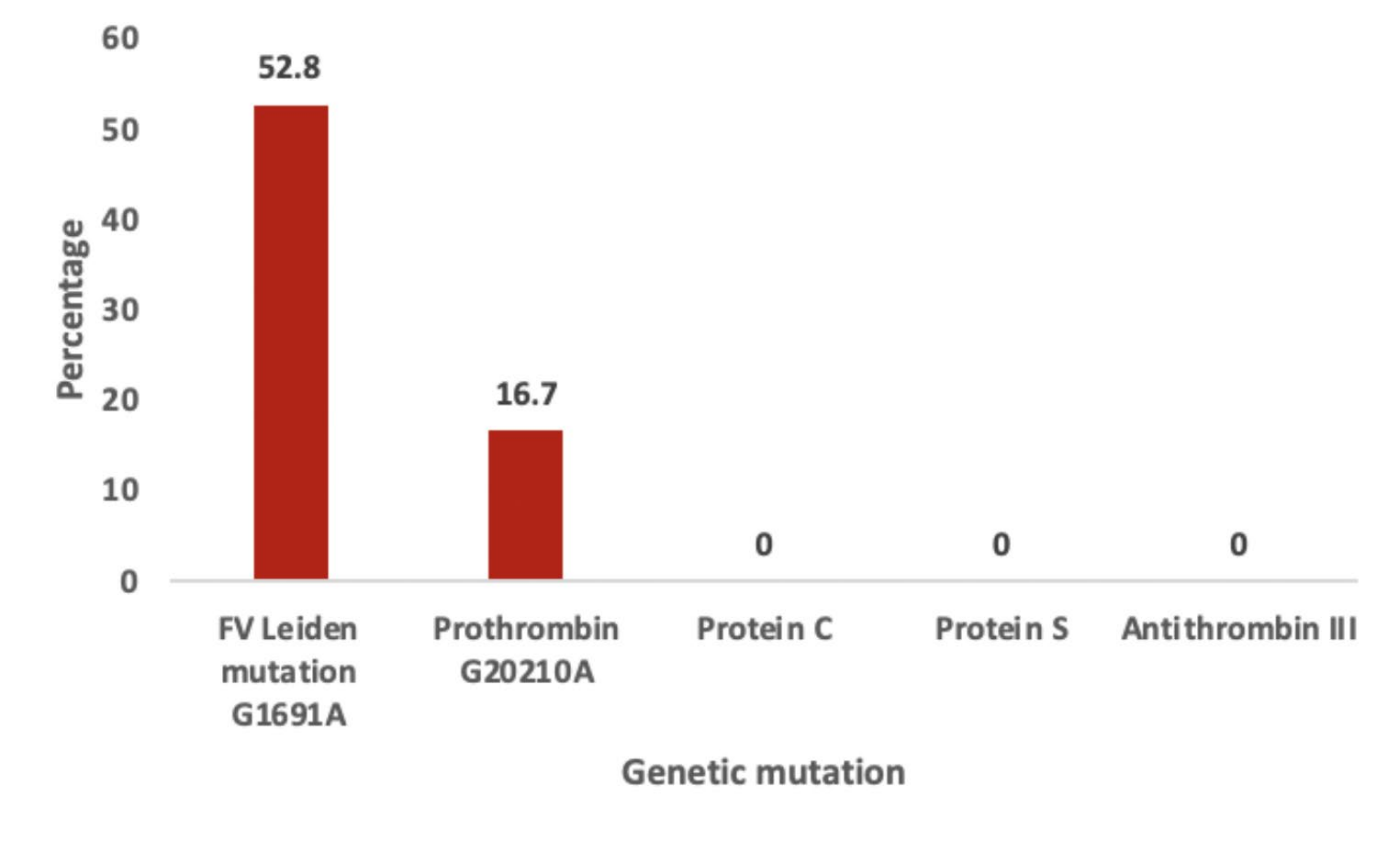
The type of gene mutations among thrombotic cases

Regarding thrombophilia gene mutation detection, 69% of patients had positive thrombophilia gene mutations (52.3% were Factor V Leiden mutation and 16.7% were prothrombin G20210A), while no mutation was detected in 31% of the studied thrombotic group. (Fig. 1)

**Fig. 2:**
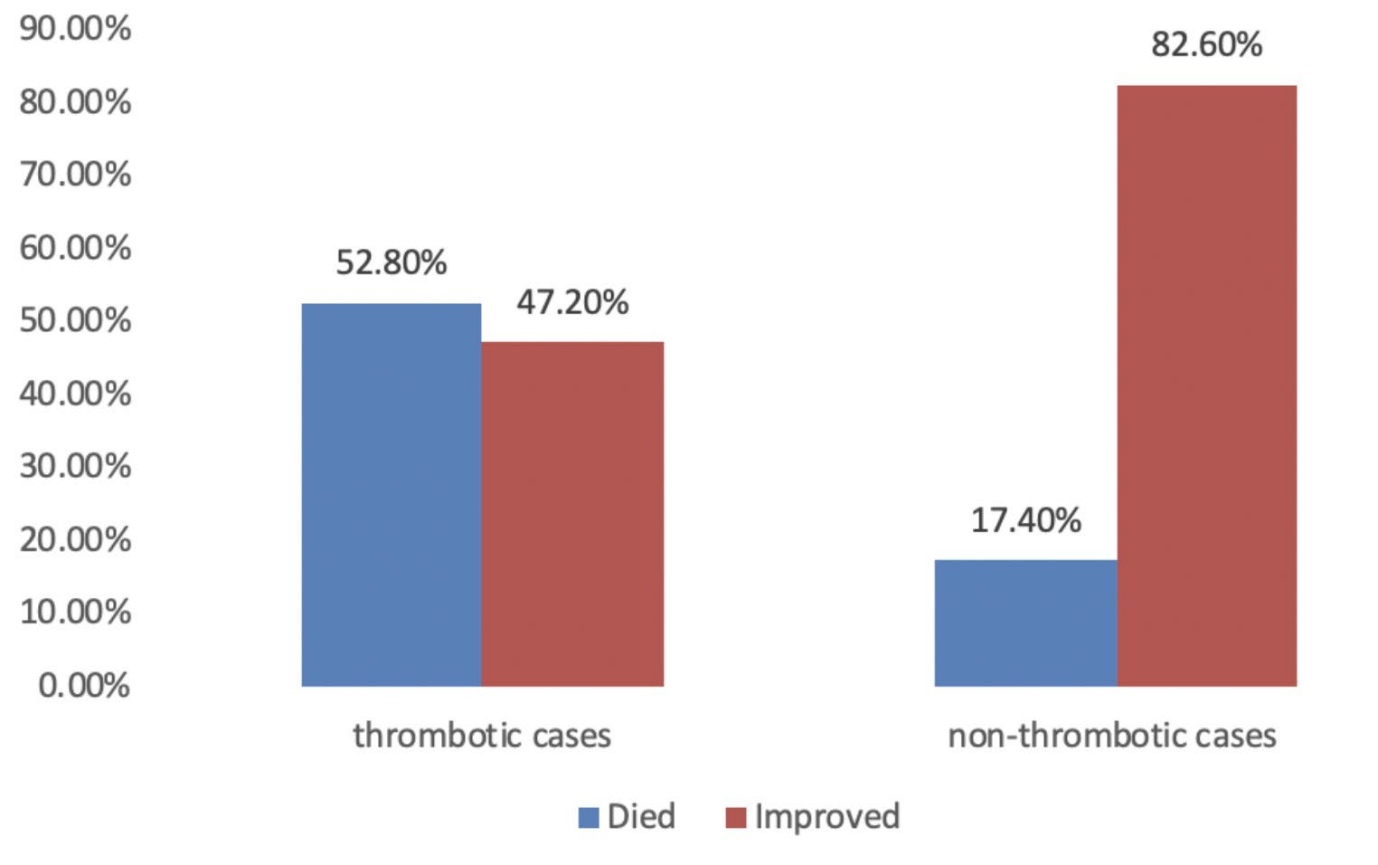
Outcome of thrombotic and non-thrombotic groups

Thrombotic patients showed increased overall mortality compared to the non-thrombotic group (52.8% vs. 17.4%; *p* < 0.001) (Fig. 2).

## Discussion

Arterial and venous thromboembolism are important causes of morbidity and mortality in neonates admitted to the NICU. Neonatal thrombotic complications were reported to be 40 times higher than other pediatric age group. This higher risk can be attributed to the exposure of the newborn to multiple risk factors [18]. Developmental hemostasis in the neonatal period is characterized by reduced fibrinolytic capacity, as proven by decreased plasminogen and increased plasminogen activator inhibitor-1, as well as lower levels of procoagulants, including factor VIII, Von Willebrand factor, and anticoagulation proteins, including protein C, protein S, antithrombin and heparin cofactor II [4]. Thrombophilia is the word used to describe coagulation problems, either acquired or genetic, that have been linked to a higher risk of thrombosis [19].

In the current study, males (55.6%) and preterm and those with low birth weight patients ( 58.3% ) showed an increased risk of thrombosis. Prematurity is a well-defined risk factor for neonatal thrombosis that increases with younger gestational age groups [20, 21]. At the same time, other studies reported that gestational age and birth weight were not associated with the risk of thrombosis [15, 22]. The family history of inherited thrombophilia was positive in only 19.4% of the thrombotic group and 10.6% of the non-thrombotic group in this study. No association between a family history of inherited thrombophilia and the incidence of neonatal thrombosis was confirmed [23, 24].

Different causes of NICU admission in thrombotic patients were detected; Respiratory distress was the most common (30.6%), while convulsions (2.7%) were the least common. However, NICU admission due to these factors carries the risk of thrombosis whatever the percentage of each one.

The duration of NICU admission was significantly higher in the thrombotic group, agreeing with the study of Bhat et al., 2018, who reported that the average length of NICU admission in thrombotic cases was about 31 days [15]. The longer the duration of the NICU stay, the more need for venous line insertion, change, pricking for sampling, and exposure to different triggering factors of thrombosis.

In this study, acquired hospital-related conditions and maternal risk factors were also associated with thrombosis risk. Maternal antenatal risk factors in the thrombotic group were variable but not significant between thrombotic and non-thrombotic patients. The association between maternal DM, hypertension, and PROM as major risk factors for neonatal thrombosis has been variably observed in multiple previous studies. Such factors alter the hemostatic balance in patients more toward hypercoagulability occurrence [19, 20, 25]. DM increases the risk of placental thrombosis which may lead to fetal embolism and thrombosis. Also, maternal hypertension and pre-eclampsia usually cause activation of the coagulation system and the development of subsequent thrombosis [19].

Vascular access is one of the most common thrombosis initiators. Mechanical and chemical damage to a vessel wall by a catheter is.

believed to initiate the thrombotic process, especially when prothrombotic risk factors are involved [20, 26–28].

The current study showed a significant difference between thrombotic and non-thrombotic cases regarding the site and duration of vascular access insertion. The longer duration of the vascular access insertion proved to be associated with an increased risk of thrombosis [15]. Longer access allows more vascular endothelial injury and more stimulation of tissue factor release, ending with thrombosis. In our study, a lower thrombosis risk was found in patients with single vascular access insertion.

Portal vein thrombosis was the most common site of neonatal thrombosis in our study (30.6%), followed by SVC thrombosis (19.4%) then DVT (13.9%), and CNS thrombosis (13.9%), about 8.3% of patients had intra-cardiac thrombosis, and only 1 patient had necrotic skin patches. About 4% (2 patients) of the enrolled patients developed thrombosis more than on-site as PVT and DVT together were reported. The use of prophylactic anticoagulation for our patients with DVT for either symptomatic or non-symptomatic cases was individualized. Due to the heterogeneity of DVT risk factors in neonates, the decision for prophylaxis for those patients was a case-by-case issue based on the standard guidelines [29]. A previous study reported that the central venous line is the most significant etiological risk factor of neonatal thrombosis [30]. while other studies proved that renal vein thrombosis, vena cava occlusion, and perinatal thromboembolic stroke are the most common thrombo-embolic events. Also, the cerebral venous sinuses, the portal, and mesenteric veins are sites of neonatal thromboembolisms with no report regarding arterial thrombosis [4, 25]. Khizroeva et al., 2023, reported that renal vein thrombosis was the most common in neonates admitted to NICU [31].

The central venous line (CVL) was associated with the highest incidence of thrombosis and no correlation between the peripheral line and Umbilical venous catheter insertion and the incidence of thrombosis [32].

Several studies reported a strong correlation between the type of vascular access and the incidence of thrombosis in neonates admitted to the NICU [18, 19]. Umbilical venous catheter (UVC) insertion is commonly associated with thrombotic cases, particularly in cases with PVT [18].

The correlation between vascular access and the incidence of thrombosis is usually explained by the complications associated with vascular access insertion, especially the development of sepsis [19].

Neonatal sepsis is the third most frequent reason for infant death. It has been associated with mortality rates of 2% in full-term infants and 20% in preterm infants. In 30% of cases, meningitis, hemodynamic and clinical instability are associated with systemic inflammatory response syndrome (SIRS), and further complications could occur according to the situation [15].

In this study, the incidence of sepsis among thrombotic patients was higher than the non-thrombotic ones. This was confirmed by higher TLC and CRP which are the precise indicators of sepsis, denoting that sepsis is an important risk factor for developing neonatal thrombosis [33]. The Variations in the definitions of infection, demographics locations, the resources available in NICUs, and infection control strategies may contribute to the variation in infection prevalence among researchers [18].

The development of thrombosis increased the mortality rate of neonates by 52.8%. Mortality was mainly associated with underlying diseases such as prematurity and sepsis rather than thrombosis per se.

Various genetic prothrombotic defects, particularly those affecting the physiological anticoagulant systems, such as antithrombin, proteins C and S deficiency, the G1691A point mutation of coagulation factor V (factor V Leiden) and the G20210A mutation of factor II are well established as risk factors for thrombotic events [4, 28, 34].

Thrombophilia gene mutation screening was performed on the thrombotic cases with a positive family history of thromboembolic events in this study and demonstrated that Factor V Leiden mutation G1691A is the most common genetic thrombophilia mutations [18] while Dugalic et al., 2021 conducted a review of gene mutations in different regions all over the world and reported that Factor V Leiden mutation and prothrombin gene mutations are very rare in the African population positive mutation has no impact on the diagnostic and treatment strategies of neonatal thrombosis [21, 35, 36]. Other reports recommended thrombophilia screening as a part of routine workup in the neonatal risk assessment of thrombosis [18].

## Conclusions

Identifying thrombosis-associated risk factors in NICU-admitted neonates is important for preventing potential thrombosis and improving the patient’s outcomes. The complexity of sepsis pathogenesis and management could potentiate multiple acquired risk factors. In the presence of familial thrombotic events, detection of inherited thrombophilia is required to prevent further morbidities.

### Limitation of the study

The unavailability of national neonatal thrombosis data to compare our results with and the lack of full data registration of enrolled patients makes it difficult to assess other probable undetected risk factors of neonatal thrombosis.

## Data Availability

No datasets were generated or analysed during the current study.

## Abbreviations

ACE I/D: Angiotensin-converting enzyme insertion-deletion
APO E: Apolipoprotein E
CADs: Central access devices
CBC: Complete blood count
CNS: Central nervous system
CRP: C reactive protein
CVL: Central venous line
DM: Diabetes mellitus
DVT: Deep venous thrombosis
FFP: Fresh frozen plasma
HTN: Hypertension
MRA: Magnetic resonance arteriography
MRI: Magnetic resonance imaging
MRV: Magnetic resonance venography
MTHFR: Methyl tetrahydrofolate Reductase
NICU: Neonatal intensive care unit
ORs: Odds ratios
PAI-1: Plasminogen activator inhibitor-1
PC: Protein c
PCR: Polymerase chain reaction
PROM: Premature rupture of membranes
PS: Protein s
PTT: Partial thromboplastin time
PVT: Portal vein thrombosis
SD: Standard deviation
SIRS: Systemic inflammatory responses
SPSS: Statistical package of social sciences
SVC: Superior vena cava
TLC: Total leucocytic count
UVC: Umbilical venous catheter
VS: Versus
